# Gender Differences in Whether and How Perceived Inequality Hampers Self-Rated Health and Mental Health: Evidence from the Chinese General Social Survey and a Randomized Experiment in China

**DOI:** 10.3390/ijerph21121640

**Published:** 2024-12-10

**Authors:** Jacqueline Chen Chen, Chenling Yu, Jianhua Zhu

**Affiliations:** School of Public Administration, Zhejing University of Technology, Hangzhou 310023, China; jcchen@zjut.edu.cn (J.C.C.); yuchenling123@163.com (C.Y.)

**Keywords:** gender differences, inequality, health, randomized experiment, China

## Abstract

A substantial body of research has explored the relationship between inequality and health, yet little is known about the gender-specific effects and pathways through which inequality affects health outcomes. This study focuses on China, a country characterized by high income inequality and uneven health distribution across social groups. In Study 1, repeated nationally representative cross-sectional data from the Chinese General Social Survey is utilized (N = 3798 for 2017, N = 1578 for 2015, and N = 2827 for 2008), revealing that perceived inequality negatively affects self-rated health, particularly among women. The high level of perceived economic inequality substantially contributes to the gender health gap in China. Study 2 employs a randomized experiment (N = 3568) to show that perceived inequality affects the health of women and men differently, that is, social mobility framing accounts for the negative effect on women’s mental well-being, whereas reducing status anxiety mainly benefits men’s mental health. To advance research on economic inequality and health, this study investigates gender differences in whether and how perceived inequality affects health.

## 1. Introduction

A significant body of literature suggests that although women tend to live longer than men, they also experience higher rates of illness [1,2]. Women’s biological strengths and healthier lifestyle choices—such as lower incidences of smoking and heavy drinking—contribute to their extended lifespans. However, factors such as lower economic status and heightened sensitivity to stressful situations and chronic stress increase their likelihood of nonfatal illnesses, leading to greater morbidity compared to men [2]. Women also spend more years in poor health than men, a disparity that has widened with the global rise in economic inequality. Nonetheless, whether the negative effect of economic inequality on health is more pronounced among women and how the mechanisms of this relationship differ between the genders remain unclear.

This study offers three main contributions. First, we conduct a randomized experiment to examine the mechanisms between economic inequality and health. Numerous studies have highlighted the negative correlation between income inequality and population health, along with the underlying mechanisms [3,4,5,6,7,8,9,10,11]. This paper provides a psychosocial perspective to understand the perception and effects of economic inequality on public health. We explore three well-known psychosocial mechanisms: social capital, status anxiety, and social mobility. These mechanisms suggest that economic inequality negatively affects health due to (1) the degradation of social connections; (2) stress from social comparisons and status competition; and (3) limited social mobility opportunities. By using different framings of economic inequality in our messages, we reveal these mechanisms. Message framing is crucial in decision-making processes [12], particularly when economic inequality is complex and has varied implications. Randomly assigning respondents to different message framings in the experiment allows us to examine the psychosocial effects of economic inequality on health and identify gender disparities.

Second, we highlight the influence of perceived economic inequality on health and investigate how this relationship unfolds within China. Economic inequality refers to the uneven distribution of valuable resources, such as wealth and income, in society. The psychosocial effects of economic inequality emerge when individuals perceive and encounter it in their daily lives. Thus, the perceived level of inequality, rather than the objective level, drives these psychosocial processes [13].

China serves as a prime example for examining the health effects of economic inequality for several reasons. First, China has one of the highest levels of economic inequality [14], with health status and problems unevenly distributed across social groups [15]. The rise in economic inequality in China has severely affected public health. Empirical studies have shown that the global trend in self-rated health (SRH) declined in China between 1990 and 2012 [15].

Second, China is maintained by structural collective mechanisms, such as geographic locations, household registration (*hukou*), and work units (*danwei)* [16,17]. Empirical evidence indicates that inequality is most prominent among different types of *danwei*; for example, workers in government agencies and public institutions earn significantly higher incomes than those in the corporate sector [18]. In addition, individuals with rural or nonlocal *hukou* face disadvantages in entering government and public institutions and accessing higher occupational opportunities [19]. Thus, economic inequality in China primarily exists at a meso-collective level rather than an individual level [16]. This often leads Chinese individuals to underestimate the relevance of economic inequality in their daily lives due to its collective nature, prompting questions about the different effects of perceived and objective economic inequality on public health in China.

Finally, we examine gender differences in whether and how perceived economic inequality affects health. Although various mechanisms have been proposed to explain the negative association between economic inequality and health, gender-specific differences in these mechanisms remain inadequately understood. With growing economic inequality, women in post-reform China face intensifying disadvantages in public and private spheres—within the labor market and the family [20,21]. Marketization has worsened gender wage gaps in urban China’s labor market due to occupational segregation [22]. According to the World Economic Forum’s Global Gender Gap Report 2023, China’s ranking in gender pay equality notably dropped from 57th in 2006 to 106th in 2023. In private settings, Chinese women also confront reproductive costs and unpaid care work due to the collapse of the *danwei* system [23].

Empirical evidence indicates that men often report better health outcomes than women across various measurements, including self-perceived well-being, illness rates, chronic disease prevalence, and overall quality of life [24,25]. This health disparity persists despite improvements in the general physical well-being of Chinese women in recent years [15,26,27]. As economic inequality and gender health disparities rise, it becomes increasingly important to explore the detrimental effects of economic inequality on health and the gender differences in these relationships.

## 2. Literature Review

A significant body of literature has explored the negative correlation and causal relationship between economic inequality and population health [3,4,5,6,7]. Individuals living in societies characterized by higher levels of inequality typically experience poorer physical and mental health compared to those in more equitable societies [15]. Empirical evidence links macro-level income inequality to reduced average life expectancy, increased mortality rates, and a higher prevalence of depression and mental illness [4,5,6,28,29,30,31]. However, controversies remain as several studies have failed to replicate these findings [32,33,34]. Psychologists attribute this inconsistency to public perceptions of economic inequality [13].

Economic inequality influences the contexts of social relationships and interactions. When individuals encounter certain levels of economic inequality in their daily lives, several psychosocial mechanisms come into play: social capital (the degree of closeness among people), status anxiety (the intensity of competition for higher social status), and social mobility (the opportunities for upward mobility). These psychosocial mechanisms on health only manifest when individuals recognize the severity of existing economic inequality. Therefore, the damaging effect of inequality is mainly driven by perceived—rather than objective—inequality [13].

This paper innovatively uses different message framings of economic inequality to represent the three mechanisms—social capital, status anxiety, and social mobility—to discuss whether and how perceived economic inequality hampers health. Message framing is a strategy for communicating complex problems in a way that the main arguments are understood and cannot be easily challenged [12]. Various studies have shown that message framing affects decision-making processes and changes people’s attitudes and behaviors [35,36]. For instance, message farming around social norms increases the payment rates for overdue taxes [37], and self-interest message framing of charitable donations encourages men with low empathy to give more [38].

In our study, we designed experimental messages to frame economic inequality in accordance with the three mechanisms. Given the diverse connotations of inequality, social capital, status anxiety, and social mobility can be elicited through different message framings. First, economic inequality is associated with the erosion of social capital and mutual trust [39]. This inequality widens the social distance between the rich and the poor, thereby leading to a lack of social cohesion and connection. Moreover, economic inequality fosters an environment where social isolation becomes more prevalent. Social isolation, defined as a lack of interaction with others, is strongly correlated with poor health conditions [40]. The mistrust and social isolation among society members lead to uncooperative behaviors, depression, and poor mental health [10,41,42]. In light of these findings, we developed a message framing of social capital that emphasizes maintaining social cohesion and trust even amid inequality. This framing highlights the crucial role social capital plays in mitigating the adverse health effects of economic disparities and fosters a more inclusive and cooperative social environment.

Second, economic inequality detrimentally affects health by increasing status competition and anxiety. As the disparity between the affluent and the less privileged widens, people gain more material benefits by outperforming their peers, which leads to increased pressure to achieve and maintain a higher social status. This pressure can manifest in various ways, such as longer working hours, heavier workloads, and fierce competition for status and recognition [43]. Moreover, in the context of economic inequality, individuals often find themselves in a constant state of comparison with others, gradually eroding self-esteem and fostering negative health behaviors. When individuals fall behind others, it can result in status anxiety, leading to higher rates of obesity, drug abuse, mental illness, and overall mortality rates [44,45]. To test the mechanism of status anxiety, we emphasize reducing social comparison and peer evaluation as this reduction helps decrease status anxiety and improve health.

Finally, the Great Gatsby Curve illustrates that economic inequality can limit individual mobility in society, thereby hampering social fairness [11,46]. Living in a highly unequal society provides the least advantaged with minimal access to economic resources and opportunities and may even result in them losing the chance of upward mobility. The inability to ascend the social ladder can create feelings of entrapment and powerlessness, resulting in diminished subjective well-being [47,48]. Given these challenges, we designed the message framing around fostering an optimistic attitude toward upward mobility. According to Hirschman’s tunnel effect, perceived mobility can provide a positive signal about a person’s prospects from a self-interest perspective [49]. This perception of mobility, even if not directly experienced, can inspire hope and encourage individuals to invest in efforts that could improve their situation, thereby mitigating the psychological harm of economic inequality.

We also examine gender differences in the negative association between inequality and health. A growing number of studies show that women are in poorer health due to their relative lack of material, personal, and social resources, along with disadvantages in using healthcare systems and vulnerability to social determinants—including social status, working hours, social support, and neighborhood environment—of health [50,51,52]. Gender differences in health can be attributed to differing structural contexts and exposure to lifestyle and psychological factors. Specifically, social structural and psychological determinants of health tend to be more important for women, whereas behavioral determinants are generally more influential for men [53]. Empirical evidence suggests that inequality has a more substantial effect on women’s health than men’s, in terms of factors such as self-reported health status, depression, and mortality [31,51,52].

Based on the literature reviewed, we develop research hypotheses for this study. Given that the psychosocial mechanisms of economic inequality depend on individuals’ perceptions of inequality, we focus on gender differences in whether and how perceived economic inequality affects SRH and mental health. Given that perceived economic inequality and the message framing of the three mechanisms are social structural and psychological determinants, we expect these factors to have a greater effect on women. Therefore, we hypothesize that women are more adversely affected by perceived economic inequality (Hypothesis 1) and that the three mechanisms—social capital, status anxiety, and social mobility—play a stronger role in the association between perceived economic inequality and women’s health (Hypothesis 2).

**Hypothesis** **1.** *The negative effect of perceived economic inequality on SRH is larger for women than men*.

**Hypothesis** **2.** *The message framings of social capital, status anxiety, and social mobility play a stronger role in relieving the detrimental effect of perceived economic inequality on health for women than on men*.

## 3. Materials and Methods

### 3.1. Study 1

#### 3.1.1. Data

To investigate gender differences in economic inequality and health, Study 1 employs data from the Chinese General Social Survey (CGSS), organized by Renmin University of China (http://cgss.ruc.edu.cn/English/Home.htm (accessed on 10 April 2024)). Launched in 2003, the CGSS is the first comprehensive, continuous, and large-scale survey project in China conducted by an academic institution. It uses probability proportional to size sampling to ensure a nationally representative sample. Each CGSS survey covers over 10,000 households from 300 to 400 villages or urban neighborhoods in 28 provinces. We selected the survey data from the years 2008, 2015, and 2017 because these included questions concerning perceived inequality. Response rates to the perceived inequality question ranged from 15% to 50%. Through a detailed selection process, we compiled a multiwave representative sample comprising 8203 respondents (3798 from 2017, 1578 from 2015, and 2827 from 2008) that meets our research criteria.

#### 3.1.2. Dependent Variable

The dependent variable is SRH, which has been consistently measured across the CGSS surveys of 2008, 2015, and 2017. This consistency enhances the reliability of cross-temporal comparisons. SRH is straightforward to measure and has been identified as a strong predictor of various health outcomes, such as mental disorders [37,54], functional ability [38,55], and mortality [39,56]. This metric is widely adopted in health science research due to its accessibility and comprehensive reflection of overall health conditions. In the three iterations of the CGSS, the respondents were asked to rate their current health using the single-item global question, “How do you rate your current physical health?” with responses ranging from “very poor” to “very good”. The responses were measured on a five-point scale, with 1 representing “very poor” and 5 representing “very good”.

#### 3.1.3. Objective and Perceived Inequality

The independent variables in this study primarily focus on income inequality, measured through objective and subjective dimensions. Objective inequality is commonly assessed using the GINI coefficient, which reflects the distribution of income within a population [6]. Given the significant developmental disparities across regions in China, this study calculates provincial-level GINI coefficients for rural and urban areas based on data from the National Bureau of Statistics of China for the survey years 2008, 2015, and 2017.

By contrast, subjective inequality is gauged through the respondents’ perceptions of inequality. In the 2008 and 2017 CGSS surveys, participants were asked to express their agreement with the statement “The income inequality in our country is too wide” on a five-point scale ranging from “strongly agree” to “strongly disagree”. The 2015 survey used a more detailed seven-point scale to capture these perceptions. To ensure consistency and comparability across the years, statistical adjustments were made to normalize the scores from these different scales, scaling them within a 0–1 range, with larger values indicating greater perceived inequality.

### 3.2. Study 2

#### 3.2.1. Experimental Design

To explore the differences between males and females in the mechanisms linking inequality and health, Study 2 employed a randomized experiment to test how different message framings of economic inequality may influence mental well-being. This experiment was conducted on “Questionnaire Star”, the largest professional online survey platform in China, with a user base exceeding 300 million. We used the platform’s sampling service to assemble a nationwide sample with a broad geographic distribution (covering all 31 provinces and 212 cities in China) and diverse socioeconomic backgrounds, including different cities, age groups, genders, educational levels, income brackets, and occupations. We set minimum and maximum time limits for completing the survey and excluded respondents who answered cheat questions incorrectly. In total, 4084 participants finished the randomized experiments, with 516 being excluded for the aforementioned reasons, resulting in a high-quality and diverse sample (N = 3568).

**Figure 1:**
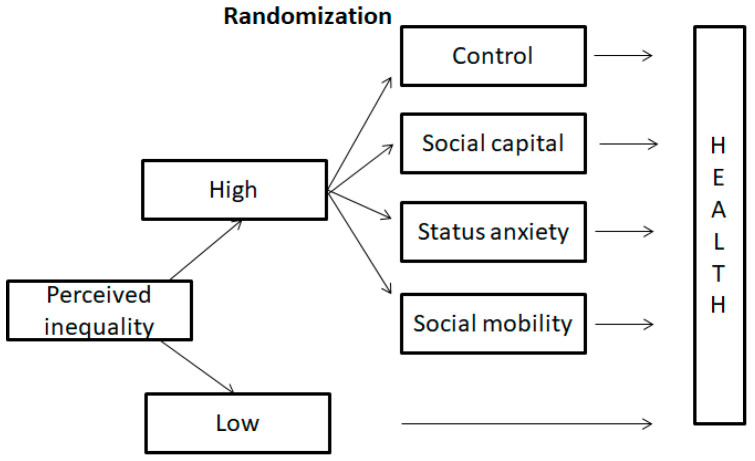
Experimental Design.

**Figure 2:**
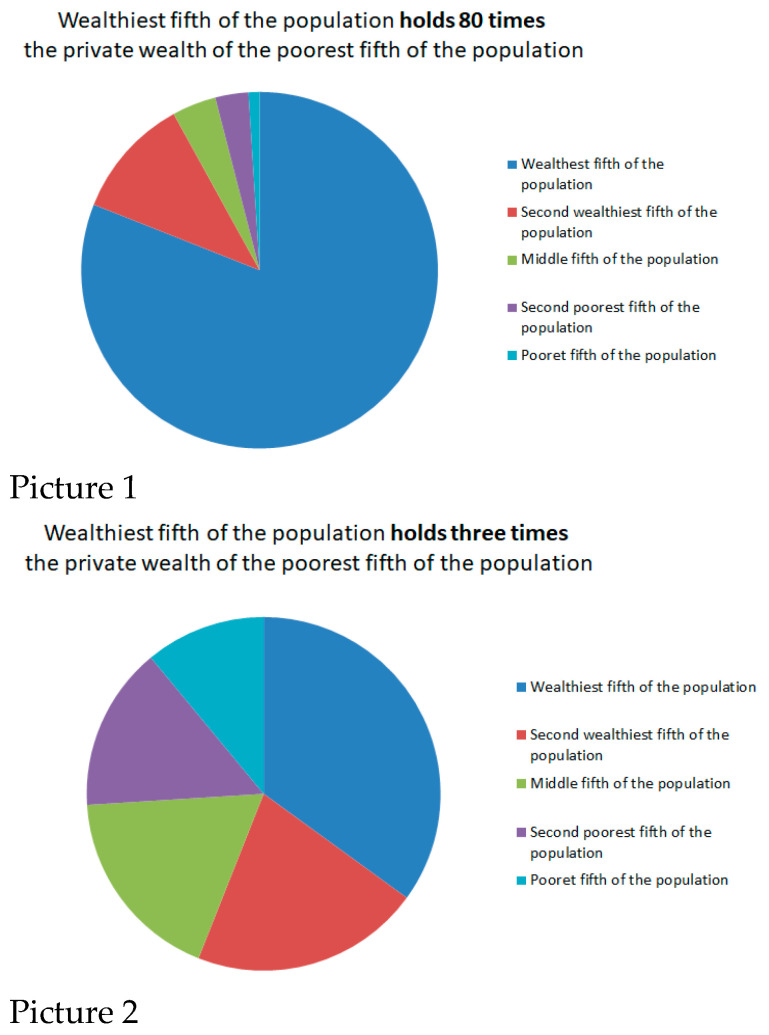
Pictures 1 and 2 in the randomized experiment.

Figure 1 presents the design of the randomized experiment. Respondents were initially asked to evaluate inequality in the city where they live. The screening question on perceived inequality was “In the following two pictures, which picture is closer to the wealth distribution in the city you live in? Two pictures show the percentage of private wealth owned by the wealthiest, second wealthiest, middle, second poorest, and poorest fifth of the population”. The respondents could choose between “Closer to picture 1” and “Closer to picture 2”. Figure 2 shows pictures 1 and 2 from the randomized experiment, representing two extreme levels of perceived inequality derived from the work of Côté et al. [57].

Once the respondents selected the perceived inequality level, those perceiving low inequality directly answered health-related questions. Participants perceiving high inequality were randomly assigned to various groups: control, social capital, status anxiety, and social mobility (distribution details in the Supplementary Materials). These respondents read differently framed messages about economic inequality before addressing the health questions. Finally, each participant completed the cheat questions and a post-experiment survey, which included demographic information.

#### 3.2.2. Message Framing of Economic Inequality

Framing significantly influences the interpretation of and reaction to messages [36,58]. We hypothesized that the effect of economic inequality on health might vary depending on the framing of the message, making the randomization of the three message framings crucial to our experiment. The survey platform required all the participants to stay on the page with the message framing for at least 20 s before proceeding. We describe the different message framings as follows.

- Control

No message was provided except, “*As noted on the previous page, there is a certain level of inequality in your city. Please click OK to complete the questions on the next page*”.

- Social Capital

Social capital refers to social cohesion and collective solidarity in a society. The first message framing was “*As noted on the previous page, there is a certain level of inequality in your city. Despite wealth disparities, society remains cohesive, and most individuals are trustworthy. Please click OK to complete the questions on the next page*”. This approach aims to reveal the positive aspects of social bonding and collective solidarity that persist even in the face of economic inequality. We considered whether a heightened sense of social capital could promote better health outcomes by providing psychological and social support while also mitigating the negative effect of perceived inequality.

- Status Anxiety

Status anxiety is the second message framing of the three groups we explored. We introduced a scenario: “*As noted on the previous page, there is a certain level of inequality in your city. Although inequality exists in society, others do not look down on me because of my job or income, nor am I expected to compare myself with others in my work. Please click OK to complete the questions on the next page*”. This narrative aims to mitigate status anxiety through a reduction in social comparison and a belief in personal competence. We wondered whether this message framing could alleviate the psychological strains of inequality.

- Social Mobility

In the social mobility scenario, the participants were presented with the following narrative: “*As noted on the previous page, there is a certain level of inequality in your city. Nevertheless, individuals from less privileged backgrounds still have opportunities to improve their lives through their own efforts Please click OK to complete the questions on the next page*”. This message underscored the fluidity of social structures and the capacity for personal effort to attain upward mobility. In this manner, we wanted to explore whether confidence in social mobility could inspire hope and motivation, thereby enhancing individuals’ mental health.

#### 3.2.3. Mental Health Scale

The online randomized experiment used the World Health Organization’s (WHO) mental health scale, known as the WHO-5 Well-being Index. This index includes five items that measure positive mood, vitality, and general interest over the past two weeks. The WHO-5 is a psychometrically sound, concise measure for evaluating psychological well-being [9]. Although it provides a general estimate of mental well-being, it has been proven effective in detecting depression within the general population [59,60]. Moreover, the cross-cultural validity of the WHO-5 enables us to estimate the mental health status of the Chinese population [61]. The five items of the WHO-5 are as follows:

I have felt cheerful and in good spirits.

I have felt calm and relaxed.

I have felt active and vigorous.

I woke up feeling fresh and rested.

My daily life has been filled with things that interest me.

The respondents chose one of the following responses that was closest to how they were feeling over the previous two weeks: “all of the time”, “most of the time”, “more than half of the time”, “less than half of the time”, “some of the time”, or “at no time”. Answers were measured by a six-point rating scale from “at no time” = 1 to “all of the time” = 6.

## 4. Results

### 4.1. Results of Study 1

Study 1 examines the effect of economic inequality on health within the Chinese population and investigates any gender differences in this relationship. The descriptive statistics for Study 1 and the control variables are provided in the Supplementary Materials. We combined multiple waves of the CGSS (2008, 2015, and 2017) and included provincial and year fixed-effects in our ordinary least squares model. In the full sample, perceived inequality is associated with poorer SRH, as indicated in Model 1, Table 1 (β = −0.198, se = 0.056, *p* < 0.001). Our test for gender heterogeneity shows that perceived inequality is not significantly associated with SRH for men in Model 3 (β = −0.054, se = 0.079, *p* = 0.4), whereas for women, perceived inequality is negatively associated with SRH in Model 5 (β = −0.347, se = 0.08, *p* < 0.001). When considering objective inequality, the actual level of inequality does not significantly affect health in the full sample (Model 2) or in the separate samples of men and women (Models 4 and 6). Generally, we found that perceived inequality, rather than objective inequality, negatively affects SRH, particularly among female respondents in China. Thus, Hypothesis 1 is validated.

### 4.2. Results of Study 2

After validating Hypothesis 1, we proceeded to test the gender differences in the mechanisms linking perceived economic inequality and health based on the randomized experiment in Study 2. Using the randomized experiment data, we found that the reliability coefficient of the WHO-5 scale is 0.9, indicating high internal validity. We averaged the WHO-5 scores to create a new variable representing mental health, where higher values denote better mental well-being. This new mental health variable served as the dependent variable in Study 2. The descriptive statistics and control variables for Study 2 are available in the Supplementary Materials.

Table 2 presents ordinary least squares regression of health on “social capital”, “status anxiety”, and “social mobility”, using the control group—individuals perceiving high inequality without message framing—as the reference. We analyzed male and female respondents in Models 1 and 2, respectively. As shown in Table 2, the male respondents are most responsive to the status anxiety message framing in Model 1 (β = 0.187, se = 0.095, *p* = 0.05), whereas the female respondents are most affected by the social mobility message framing in Model 2 (β = 0.202, se = 0.087, *p* = 0.021).

We examined how socioeconomic status (SES) estimates influence mental health. Table 2 indicates that respondents with more years of education and higher social status tend to report better mental health, regardless of gender. Higher social positions enhance access to social resources and provide protection against social risks—such as poverty, unemployment, and homelessness—that can negatively affect mental health. In addition, our findings reveal that years of education and high social status have a more pronounced effect on women than on men, emphasizing the greater influence of social structural determinants on women’s health [50,51,53].

**Figure 3:**
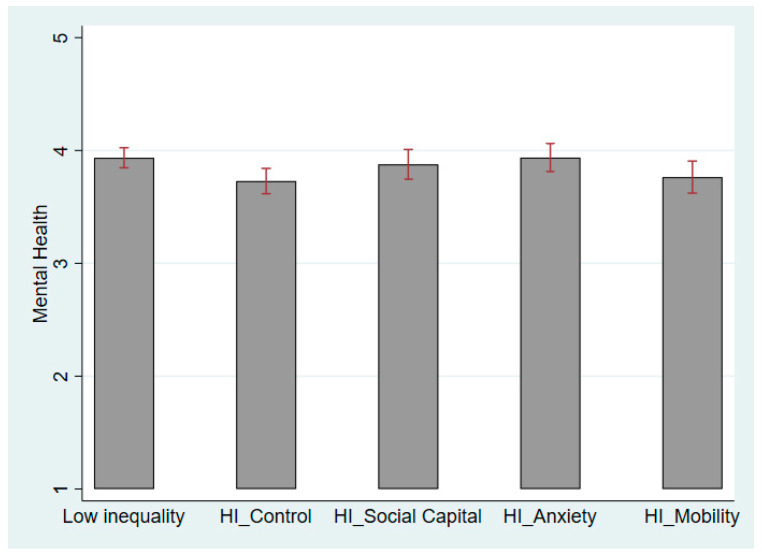
Mental Health in Low- and High-Inequality Scenarios for Male Respondents. Note: HI is short for High Inequality.

**Figure 4:**
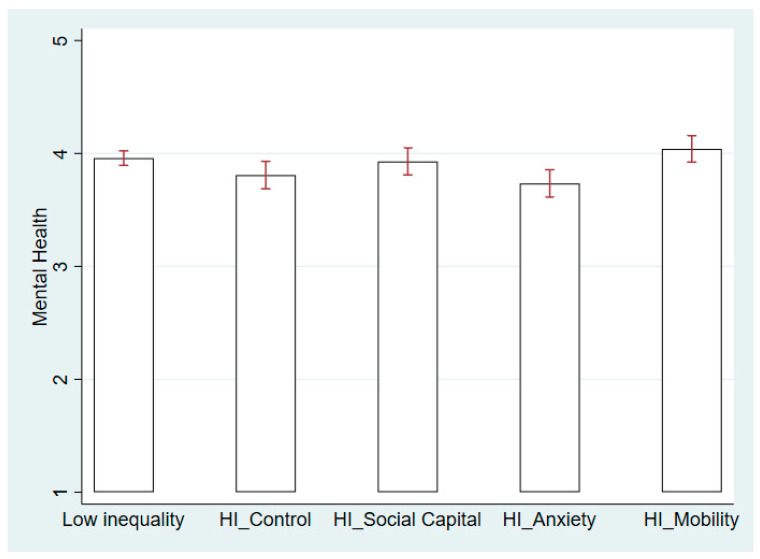
Mental Health in Low- and High-Inequality Scenarios for Female Respondents. Note: HI is short for High Inequality.

Figure 3 and Figure 4 further illustrate how different message framings contribute to changes in mental well-being among male and female respondents. When men experience high economic inequality, a reduction in status anxiety and social comparison helps maintain better mental well-being. Therefore, the status anxiety message framing significantly improves men’s mental health, as shown in Figure 3. For the male respondents, the difference in health between the status anxiety group and the low inequality group is insignificant. By contrast, women did not react to the status anxiety message framing in Model 2 of Table 2. The female respondents may improve their mental health if they believe that social mobility is guaranteed under high perceived economic inequality. Figure 4 shows that the effect of the social mobility message even surpasses the level of mental health for women in the low-inequality scenario. Thus, we observe apparent gender differences in the mechanisms linking perceived inequality and health, that is, status anxiety affects the male respondents’ health more, whereas concerns about social mobility affect female respondents more.

In the social capital group, the male and female respondents reported better mental health when exposed to the message framing of social capital (Figure 3 and Figure 4), yet this increase showed no gender difference. Therefore, we consider that no gender difference exists in the social capital mechanism linking perceived economic inequality and health. The lack of social capital and mutual trust harms the health of men and women similarly [5]. Hypothesis 2 is thus partially confirmed.

## 5. Discussion

This study examines gender differences in the negative association between perceived economic inequality and health in the Chinese context. First, women’s health suffers from perceived inequality, which is consistent with previous studies on women’s vulnerability to economic inequality [50,53]. We also discovered that the framing of social mobility messages significantly mitigates the negative effect of perceived inequality on women’s health as compared to men. According to Hirschman’s tunnel effect hypothesis, individuals tolerate economic inequality if it signals positive prospects for themselves [49]. We found that women are more responsive to the tunnel effect than men. Women have relatively limited access to economic and social resources [62] and are more vulnerable to material, behavioral, and psychological conditions influencing health [50]. Nevertheless, belief in social mobility buffers the negative effect of perceived inequality on women’s health, indicating potential policy implications for improving women’s health.

Second, by using multiwave nationwide surveys in Study 1, we discovered that men’s health is not significantly influenced by either objective or perceived inequality. However, Study 2 reveals that message framing, which reduces status anxiety, is more effective in improving men’s health when they perceive high economic inequality. Although existing literature suggests that women globally have a higher prevalence of social anxiety than men [63], reducing status anxiety benefits men’s health more in China. This might be explained by differences in self-construal; in Western cultures, men usually develop an independent self-construal, whereas women develop an interdependent one [64]. This interdependence leads to social comparison and greater susceptibility to social anxiety. However, in East Asian cultures, all individuals generally have a higher interdependent social construal [65], resulting in comparable levels of social anxiety among Chinese men and women [66].

In addition, the participants in the randomized experiment were relatively young (mean age = 33), compared to the national survey sample (mean age = 48). By randomly assigning respondents to different treatment groups, we removed the endogeneity issues related to the three mechanisms and health. Nonetheless, the experimental findings are based on a younger demographic. In Chinese societies, due to patrilineal traditions, young men often bear the financial responsibilities related to marriage, such as housing, bride price, and the cost of the marriage banquet [67,68], leading to increased financial burdens and social comparison. This intensifies status competition and social anxiety among young Chinese men. Thus, we discovered that reducing status anxiety alleviates the detrimental effect of perceived economic inequality on health, particularly among young Chinese men.

Finally, we propose that future studies on economic inequality and health should attach more importance to perceived inequality, as individuals’ perceptions primarily influence the psychological effects of inequality [13]. The lives of the rich and poor are increasingly segregated by distinct institutions, resulting in interactions primarily among individuals of similar education and income levels [69]. This phenomenon contributes to a significant underestimation of the true extent of inequality [70,71]. In China, the gap between objective and perceived inequality is particularly pronounced due to structural collective mechanisms that perpetuate inequality [16,17]. Moreover, the government’s control over the media through propaganda and censorship leads to misinformation about economic inequality [72]. Consequently, perceived inequality, rather than objective inequality, may be more relevant when examining the effect of inequality on public well-being in China.

This study highlights the importance of perceived inequality on health in China, without undermining the significance of objective inequality. Objective measures of inequality are essential for identifying broader societal disparities that contribute to poorer living conditions and increased health risks [3,4,5,6,7]. Perceived and objective inequalities are crucial for understanding their combined effects on health and well-being. Perceived inequality offers insight into individuals’ psychological experiences, a key determinant of health outcomes, while objective inequality reflects the systemic challenges faced by individuals.

Although this study offers valuable insights into gender differences in whether and how perceived economic inequality affects health, it has certain limitations. First, the data and randomized experiments are specific to China, which may limit the generalizability of our findings to other cultural and societal contexts. This focus was chosen due to the availability of comprehensive data and the unique socio-economic dynamics within China. Future research should aim to conduct cross-cultural comparisons across different countries with diverse economic structures and cultural backgrounds.

Second, the CGSS data utilized in our study rely on SRH measures and lack objective health status indicators. Consequently, our findings are based on self-assessed health rather than objective health metrics. Similarly, our assessment of economic inequality faces a comparable limitation, as the CGSS includes only a single-item question regarding perceived income inequality. Future research should incorporate a broader range of health indicators and measures of economic inequality to achieve a more comprehensive understanding of the health impacts of economic inequality.

Lastly, our study focuses solely on the immediate effects of message framing on self-rated and mental health, without considering potential long-term effects. Future research employing longitudinal designs could provide valuable insights into whether the effects of specific message framing on health are sustained over time.

## 6. Conclusions

This study explores gender differences in how economic inequality negatively affects health and its underlying mechanisms. We find that women’s health is more adversely affected by perceived economic inequality, and framing messages around upward mobility can help mitigate these effects for women. By contrast, men are less affected by perceived inequality but are more sensitive to status competition, with reduced status anxiety helping to diminish the adverse effect of inequality on their health.

The study carries several practical implications. Perceived economic inequality detrimentally affects population health, particularly among women. More importantly, the psychological consequences of economic inequality vary based on its message framing. Even when individuals experience similar inequality levels, different messaging about inequality leads to significant gender differences in mental well-being. Promoting a belief in social mobility can enhance women’s well-being, whereas reducing status anxiety can improve health outcomes for men. To mitigate the detrimental effect of economic inequality on health, policymakers may consider employing effective message-framing strategies.

Although appropriately framing inequality messages is essential for public health improvement, we must not disregard the well-established negative relationship between objective economic inequality and public health, as supported by extensive research over the decades [3,4,5,6,7]. Studies have also shown that objective inequality negatively affects health in China [73,74,75], one of the world’s most unequal societies. Despite various initiatives by the Chinese government to reduce inequality, it remains high and is likely to continue. We suggest that future studies on inequality and health in China should consider objective measures of inequality, such as the Gini and Theil coefficients, and perceived inequality. Furthermore, investigating how inequality is created and maintained in China and how individuals perceive it in their daily lives can provide a more comprehensive approach to addressing health disparities in China and other unequal societies.

## Figures and Tables

**Table 1:** OLS Regression of SRH on Objective and Perceived Economic Inequality.

	Model 1	Model 2	Model 3	Model 4	Model 5	Model 6
Sample	Full sample	Full sample	Male	Male	Female	Female
Perceived inequality	−0.198 ***		−0.054		−0.347 ***	
	(0.056)		(0.079)		(0.080)	
Objective inequality		−0.347		−0.148		−0.524
		(0.294)		(0.429)		(0.407)
Individual-level control variables						
Woman	−0.147 ***	−0.144 ***				
	(0.022)	(0.022)				
Age	−0.033 ***	−0.034 ***	−0.023 ***	−0.023 ***	−0.043 ***	−0.045 ***
	(0.004)	(0.004)	(0.005)	(0.005)	(0.005)	(0.005)
Age square	0.000 **	0.000 ***	0.000	0.000	0.000 ***	0.000 ***
	(0.000)	(0.000)	(0.000)	(0.000)	(0.000)	(0.000)
Education years	0.021 ***	0.021 ***	0.016 ***	0.016 ***	0.023 ***	0.023 ***
	(0.003)	(0.003)	(0.005)	(0.005)	(0.004)	(0.004)
Family income (ln)	0.060 ***	0.060 ***	0.063 ***	0.063 ***	0.060 ***	0.060 ***
	(0.007)	(0.007)	(0.010)	(0.010)	(0.009)	(0.009)
Urban	0.099 ***	0.097 ***	0.064	0.063	0.130 ***	0.128 ***
	(0.026)	(0.026)	(0.037)	(0.037)	(0.037)	(0.037)
High SES	0.242 ***	0.160 ***	0.210 ***	0.174 ***	0.270 ***	0.145 ***
	(0.040)	(0.022)	(0.056)	(0.032)	(0.057)	(0.033)
Province-level control variables						
GDP per capita (ln)	0.196	0.049	(0.037)	−0.189	0.146	0.169
	(0.193)	(0.273)	0.191	(0.392)	(0.269)	(0.381)
Disposable income	0.545	0.424	0.212	1.075	1.117	−0.016
	(0.551)	(0.558)	(0.776)	(0.795)	(0.784)	(0.787)
Province fixed-effect	Yes	Yes	Yes	Yes	Yes	Yes
Year fixed-effect	Yes	Yes	Yes	Yes	Yes	Yes
Constant	−1.315	−3.939	−6.873	−3.764	2.018	−4.393
	(5.150)	(3.100)	(7.329)	(4.459)	(7.261)	(4.312)
R-square	0.217	0.216	0.197	0.197	0.233	0.230
N	8.203	8.203	3.970	3.970	4.233	4.233

Note: *** *p* < 0.001, ** *p* < 0.01.

**Table 2:** OLS Regression of Mental Health on Different Experimental Groups.

	Model 1	Model 2
Sample	Male	Female
High inequality no message (reference group)		
High inequality social capital	0.133	0.120
	(0.095)	(0.086)
High inequality status anxiety	0.187 *	−0.099
	(0.095)	(0.087)
High inequality social mobility	−0.006	0.202 *
	(0.095)	(0.087)
Low inequality	0.204 *	0.141 *
	(0.080)	(0.070)
Control variables		
Age	−0.025	0.019
	(0.020)	(0.018)
Age square	0.000	−0.000
	(0.000)	(0.000)
Education years	0.040 *	0.051 **
	(0.019)	(0.020)
High socio-economic status	0.147 *	0.239 ***
	(0.071)	(0.059)
Income (ln)	0.041	0.014
	(0.031)	(0.025)
Constant	3.278 ***	2.472 ***
	(0.441)	(0.431)
R-squared	0.049	0.045
N	1.568	2.000

Note: *** *p* < 0.001, ** *p* < 0.01, * *p* < 0.05.

## Data Availability

Original experimental data can be shared in the Supplementary Materials.

## Supplementary Materials

The following supporting information can be downloaded at: https://www.mdpi.com/article/10.3390/ijerph21121640/s1, Table S1. Distribution of Participants in Randomized Experiment; Table S2. Descriptive Statistics of CGSS 2008, 2015, 2017 (Study 1); Table S3. Descriptive Statistics of Randomized Experiment (Study 2, N = 3568).
