# Supplementary material for: Gender Differences in Whether and How Perceived Inequality Hampers Self-Rated Health and Mental Health: Evidence from the Chinese General Social Survey and a Randomized Experiment in China
Source: Int J Environ Res Public Health. 2024 Dec 10;21(12):1640. doi: 10.3390/ijerph21121640 (PMC11675398; doi:10.3390/ijerph21121640)
Supplement: Supplementary file 1 [file ijerph-21-01640-s001.zip › ijerph-3224440-supplementary1209.pdf]

International Journal of
Environmental Research and Public Health

MDPI

Supplementary Materials:

1. The distribution of participants in each group

Table S1 shows the participant distribution across each group in the randomized experiment. The "control", "social capital", "status anxiety", and "social mobility" groups comprised 13.73%, 14.24%, 14.18%, and 14.18% of the sample, with 490, 508, 506, and 506 participants in each group, respectively. The low-inequality group included 43.67% of the participants, totaling 3568 individuals.

Table S1. Distribution of Participants in Randomized Experiment

Group	Frequency	Percentage (%)
Control	490	13.73
Social capital	508	14.24
Status anxiety	506	14.18
Mobility	506	14.18
Low inequality	1558	43.67
Total	3568	100

2. Descriptive statistics for Studies 1 and 2

Table S2. Descriptive Statistics of CGSS 2008, 2015, 2017 (Study 1)

Variable	Numbers	Mean	Standard Deviation	Minimum	Maximum
Self-rated health	8203	3.57	1.08	1	5
Perceived inequality	8203	0.80	0.20	0	1
GINI coefficient	8203	0.43	0.04	0.35	0.51
Individual-level control variables					
Woman	8203	0.52	0.5	0	1
Age	8203	48.05	16.12	18	103
Education years	8203	9.1	4.536	0	19
Urban	8203	0.63	0.48	0	1
Family income(ln)	8203	10.138	1.79	0	16.12
High SES	8203	0.080	0.27	0	1
Province-level control variables					
GDP per capita(ln)	8203	1.64	0.45	0.68	2.68
Disposable income	8203	20595.98	11358.42	5533	57230

Table S3. Descriptive Statistics of Randomized Experiment (Study 2, N= 3568)

Variable	Note	Mean	Standard Deviation	Minimum	Maximum
Mental health					
Mental health 1	At no time=1, All of the time=6	4.03	1.29	1	6
Mental health 2		4.05	1.26	1	6
Mental health 3		3.88	1.30	1	6
Mental health 4		3.85	1.32	1	6
Mental health 5		3.67	1.23	1	6
Individual-level control variables					
Woman	Male = 0, Female = 1	0.56	0.50	0	1

Age		32.52	7.96	18	65
Education years		15.76	1.33	0	18
Yearly income (ln)		11.38	1.04	8.52	13.30
High socio-economic status (SES)	Middle and low SES = 0, High SES =1	0.19	0.39	0	1

3. Control variables for Studies 1 and 2

In Study 1, we incorporated several control variables that may confound the relationship between inequality and health, following common practice in previous studies. These variables include gender, urban/rural status, years of education, family income, SES, age, and age squared. All are continuous variables, except for gender, urban/rural classification, and high SES. SES, income, and years of education reflect developmental opportunities and access to resources, factors known to be associated with health outcomes. Age and age squared are included to capture any potential nonlinear relationship between age and health. Furthermore, we control for province-level Gross Domestic Product (GDP) per capita (ln) and disposable income to eliminate the effect of regional development on health outcomes. In Study 2, we control for years of education, yearly income, high SES, age, and age squared. Given that yearly income is a categorical variable, we include an income fixed-effect in the model.
